# Radiographic findings in patients suspected of subacromial impingement syndrome in relation to shoulder pain and disability

**DOI:** 10.1007/s00256-025-04916-3

**Published:** 2025-04-23

**Authors:** Linda Christie Andrea, Susanne Wulff Svendsen, Poul Frost, Kate Smidt, John Gelineck, David Høyrup Christiansen, Søren Rasmussen Deutch, Torben Bæk Hansen, Annett Dalbøge

**Affiliations:** 1 Department of Radiology, Gødstrup Hospital, Aarhus, Denmark; 2 Department of Occupational Medicine, Danish Ramazzini Centre, University Research Clinic, Gødstrup Hospital, Herning, Denmark; 3 Department of Occupational Medicine, Danish Ramazzini Centre, Aarhus University Hospital, Aarhus, Denmark; 4 Department of Orthopedic Surgery, Viborg Regional Hospital, Viborg, Denmark; 5 Department of Radiology, Aarhus University Hospital, Aarhus, Denmark; 6 Department of Clinical Medicine, Health, Aarhus University, Aarhus, Denmark; 7 Elective Surgery Centre, Silkeborg Regional Hospital, Silkeborg, Denmark; 8 Centre for Research in Health and Nursing, Regional Hospital Central Jutland, Viborg, Denmark; 9 Department of Orthopedic Surgery, Randers Regional Hospital, Randers, Denmark; 10 Hip and Knee Surgery, University Clinic of Hand, Gødstrup Hospital, Herning, Denmark

**Keywords:** Impingement, Subacromial pain, Radiograph, Shoulder, Oxford shoulder score

## Abstract

**Objective:**

The aim was to study the association between specific radiographic findings and patient reported shoulder pain and disability in patients suspected of subacromial impingement syndrome (SIS).

**Materials and methods:**

This cross-sectional study used baseline data from a prospective study. Study population included patients age 18 to 63 years, referred to orthopaedic clinic on suspicion of SIS. Radiographic findings before first visit to a department of orthopaedic surgery comprised subacromial calcifications, acromial morphological characteristics (i.e. acromial type and spur), acromioclavicular osteoarthritis, signs of previous glenohumeral dislocation (Bankart/Hill-Sachs lesions), and architectural measures (i.e. acromial tilt, acromion index, and lateral acromial angle). Shoulder pain and disability were evaluated using the Oxford Shoulder Score (OSS) from patient’s response to a questionnaire at first visit to the public department of orthopaedic surgery or before surgery for SIS. A low OSS was defined as having a score < 25. Associations between the radiographic findings and low OSS were analysed using logistic regression.

**Results:**

The population comprised 825 patients. Median time between radiographic examination and completion of the questionnaire was 9 days (*SD* = 27.1). In adjusted analysis, we found a statistically significant association for lateral spur especially birdbeak type (*OR* = 2.24 (95% CI 1.36–3.71)), Bankart/Hill-Sachs lesion (*OR* = 2.49 (95% CI 1.38–4.48)), and acromial tilt > 35° (*OR* = 0.62 (95% CI 0.41–0.94)). Female sex (*OR* = 2.25 (95% CI 1.59–3.18)) was also associated with low OSS.

**Conclusion:**

In terms of associations with patient-reported shoulder pain and disability, lateral spurs, with emphasis on birdbeak type, Bankart/Hill-Sachs lesions, and acromial tilt > 35°, seemed clinically important.

## Introduction

In patients evaluated for subacromial impingement syndrome (SIS), radiographic findings of presumed clinical importance include subacromial calcification [1–7], acromial morphological characteristics (e.g. hooked acromion or spurs) [2–6, 8–10], acromioclavicular osteoarthritis (OA) [3, 4, 6, 8, 11], and previous glenohumeral (GH) dislocation of the glenohumeral (e.g. Bankart and/or Hill-Sachs lesion) [12]. In patients referred to a Danish public department of orthopaedic surgery on suspicion of SIS, the overall prevalence was 24.4% for calcification, 15.8% for hooked acromion, 11.1% for lateral acromial spurs, 6.6% for medial acromial spurs, 12.0% for acromioclavicular OA, and 7.1% for Bankart and/or Hill-Sachs lesions [13].

In Denmark, routine radiographic examination is performed before first evaluation at an orthopaedic department on suspicion of SIS. For radiographic findings to be relevant with respect to diagnosis, choice of treatment, and inference of prognosis, the findings must play a role for the patients’ symptoms. Patients with shoulder pain often have no pathologic radiographic findings [3, 7, 14, 15], whereas a high prevalence of radiographic pathologies has been reported among patients with asymptomatic shoulders [7, 14, 15]. Together, this shows that the patients’ symptoms may be unconnected with the findings. Regarding response to treatment, radiographic imaging in combination with ultrasound examination did not improve the ability to rule-out a positive response to a diagnostic injection of local anaesthetic into the subacromial space in primary care patients presenting to their practitioner with a new episode of shoulder pain [4]. Likewise, radiographic findings of calcification, acromial spurs, and acromioclavicular OA have shown no correlation to the short-term outcome of medical treatment of shoulder pain with injections of local anaesthetics into the subacromial space or orally administered non-steroid anti-inflammatory drugs among patients with pain during abduction of humerus [8]. Finally, size, localisation, and type of calcification do not seem to correlate with the results of non-surgical treatment of patients with calcifications [16]. A recent systematic review underlined the need for further research into relationships between radiographic findings and shoulder symptoms [17]. We have not identified studies that have evaluated associations between specific radiographic findings and patient-reported shoulder pain and disability in patients examined on suspicion of SIS.

The aim of the present study was to evaluate the association between radiographic findings with reference to subacromial calcifications, acromial morphological characteristics, acromioclavicular OA, previous trauma, and architectural measures (i.e. acromial tilt, acromion index, and lateral acromial angle) and patient-reported shoulder pain and disability in patients examined on suspicion of SIS. We hypothesised that the presence of calcification, a Bigliani type III (hooked) acromion, acromial spurs, acromioclavicular OA, a low acromial tilt, and a low lateral acromial angle were associated with poorer patient-reported shoulder pain and disability.

## Materials and methods

### Design and population

We conducted a cross-sectional study using baseline data from a prospective study of shoulder patients in Central Denmark Region [18]. The study population has previously been described [13]. In brief, the population included patients, referred to one of six public departments of orthopaedic surgery on suspicion of SIS in the period 1 January 2011 to 28 February 2012. Inclusion criteria was age 18–63 years, residence in 18 out of 19 municipalities in Central Denmark Region (an island municipality was left out), at least one visit to a public department of orthopaedic surgery, response to a questionnaire at first visit or before surgery for SIS, and at least one available radiograph of the shoulder [13]. We excluded patients that did not fulfil the questionnaire.

The study was authorised by the Danish Data Protection Agency (journal number 2010–41–4316) and the Danish National Board of Health permitted the evaluation of radiographic examinations (reference number 3–3013-192/1/). In Denmark, questionnaire and register studies do not require approval by committees on health research ethics.

### Oxford shoulder score

Oxford Shoulder Score (OSS) is a 12-item outcome measure of patient-reported shoulder pain and disability ranging from 0 (worst) to 48 (best) points [19, 20]. Data on OSS were extracted from patients’ response to a questionnaire at first visit at a public department of orthopaedic surgery or before any surgery for SIS. Participants were asked to consider their most painful shoulder when answering the OSS, or, in case of same level of pain, the right shoulder. We defined a low OSS as < 25 points, thus focusing on the third of the patients with more severe symptoms.

### Radiographic examination

**Fig. 1:**
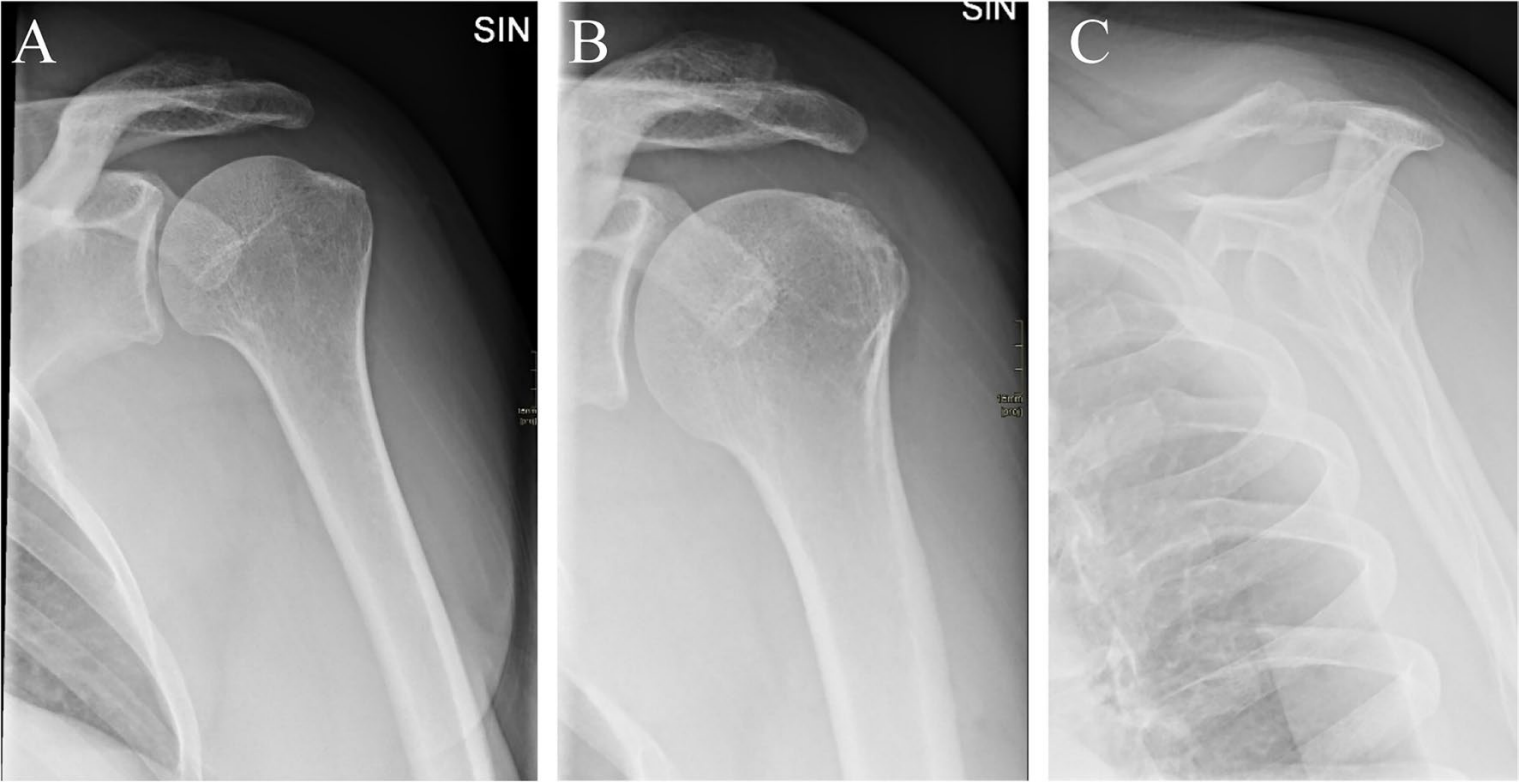
Standard projections. Anterior–posterior projections with humerus in external rotation (**A**), internal rotation (**B**), and outlet view (**C**)

Radiographic examination was routinely performed before the first visit to a public department of orthopaedic surgery. We have previously described the radiographic examinations. In brief, the examinations included up to three radiographs i.e. anterior–posterior (AP) views in external and internal rotation and an outlet view (i.e. lateral projection with 10°–20° cranio-caudal angulation of the ray) (Fig. 1). The evaluation of the radiographs was performed by two medical doctors at residential level of orthopaedic education (LCA and KS), supervised by an experienced musculoskeletal radiologist (JG). The evaluation was done using a detailed manual with extensive illustrations, and an initial procedure of calibration between the two evaluators was performed [13].

**Fig. 2:**
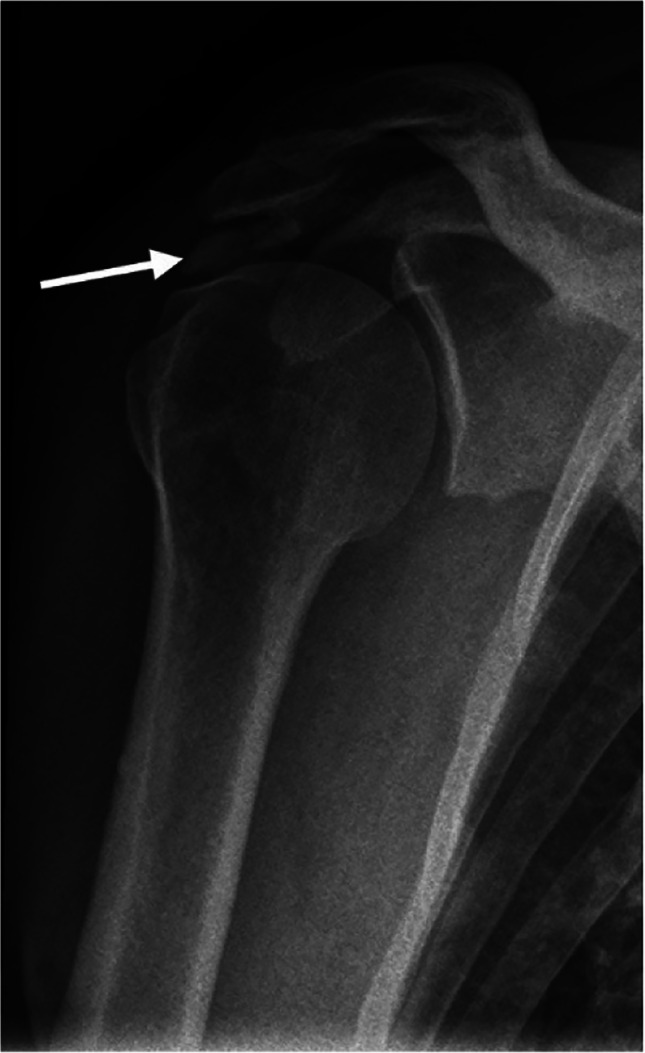
Subacromial calcification

**Fig. 3:**
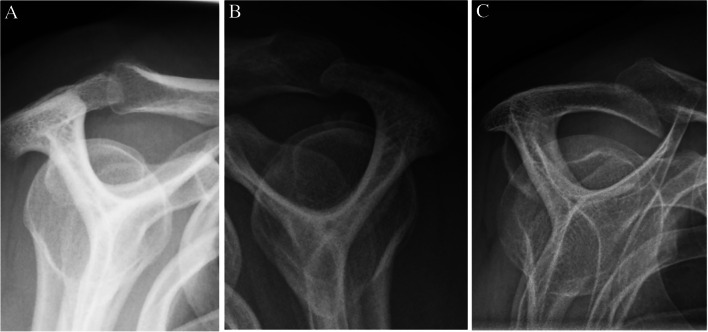
Acromion types: type I, flat (**A**); type II, curved (**B**); type III, hooked (**C**)

**Fig. 4:**
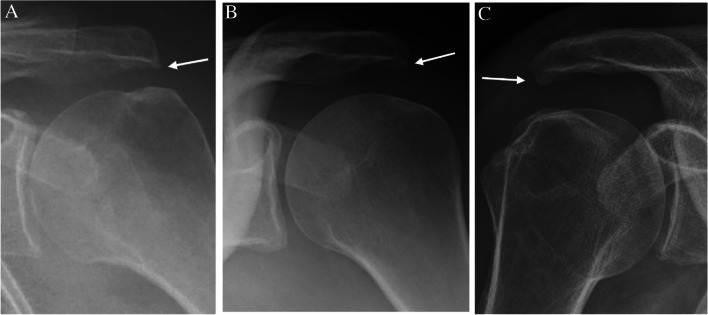
Lateral spurs in three different patients: bird beak type (**A** + **B**) and heel type (**C**)

**Fig. 5:**
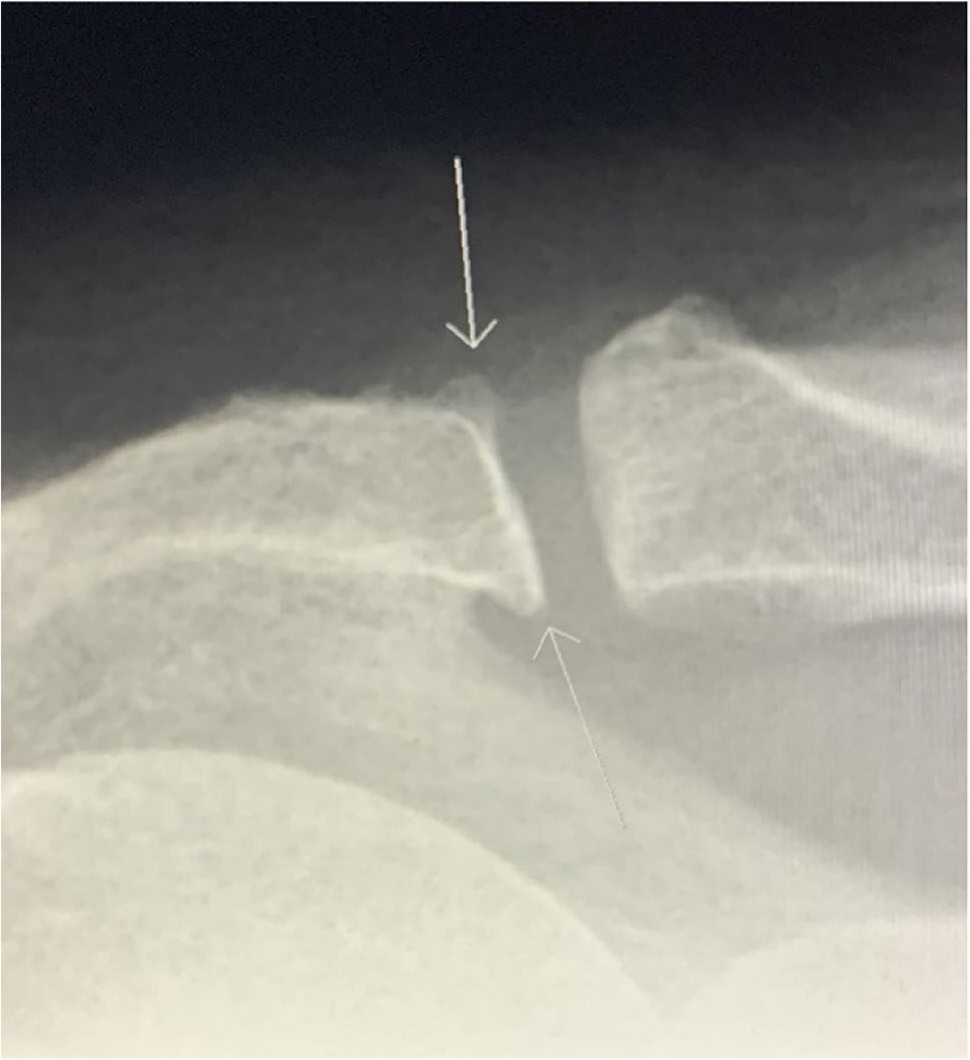
Medial acromion spurs

**Fig. 6:**
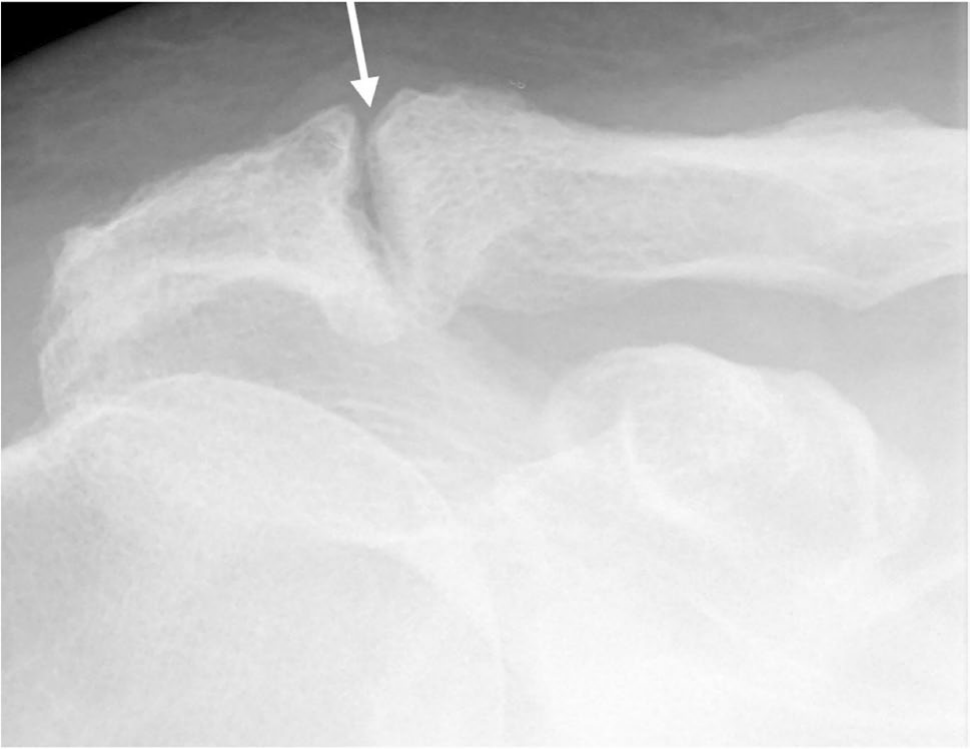
Osteoarthritis of the acromioclavicular joint with narrowing of joint and spurring of medial acromion

**Fig. 7:**
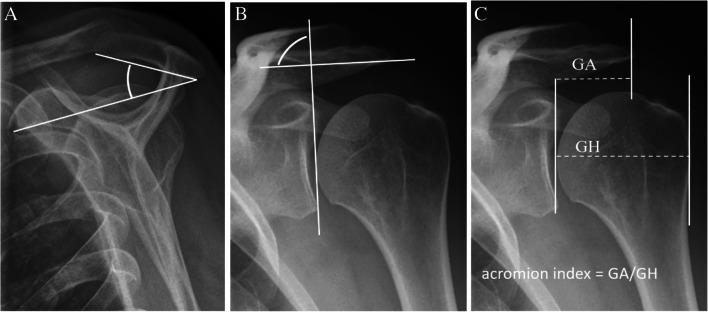
Acromial tilt (**A**). Lateral acromial angle (**B**). Acromion index (**C**)

The radiographic findings comprised subacromial calcifications (Fig. 2), acromial morphological characteristics, acromioclavicular OA, signs of previous GH dislocation (Bankart and/or Hill-Sachs lesions), and architectural measures (i.e. acromial tilt, acromion index, and lateral acromial angle). For subacromial calcifications, we included presence (no/yes), area (no calcification, ≤ 0.2 cm^2^, > 0.2 to ≤ 0.6 cm^2^, and > 0.6 cm^2^), and characteristics according to Molés classification (no calcification, types A and B, type C, and type D) of any calcifications. No differentiation of the observed calcifications, based on the suspected underlying pathology of calcifications, was done (e.g. calcific tendinitis or CPPD). For acromial morphological characteristics, we included the Bigliani classification of acromion shape (type I “flat”, type II “curved”, and type III “hooked”) (Fig. 3); the presence (no/yes); types of lateral spurs (i.e. no lateral spur, heel type, traction type, and birdpeak type) (Fig. 4); and presence of medial acromial spurs (no/yes) (Fig. 5). For acromioclavicular OA, we included the presence (yes/no) (Fig. 6). For signs of previous GH dislocation, we focused on the presence of either Bankart or Hills Sachs lesion (yes/no). For the architectural measures, we included acromial tilt (angle between undersurface of acromion and line from tip of coracoid process to posterior aspect of acromion), acromion index (relationship between distance from glenoid fossa to lateral aspect of acromion resp. humerus), and lateral acromial angle (angle between acromion undersurface and glenoid fossa) (Fig. 7) [13]. The architectural measures were categorised into 3 groups (i.e. acromial tilt: 13.6° to 30°, > 30°to 35°, and > 35° to 50.7°, acromion index: 0.23 to 0.6, > 0.6 to 0.7, and > 0.7 to 0.96, and lateral acromial angle: 50.3° to 80°, > 80° to 90°, and > 90° to 121°). We used IMPAX version 6.5 to evaluate the radiographs, including in-programme tools to measure calcification areas as well as angles and distances for architectural measures.

### Covariates

Covariates included sex, age, duration of patient reported shoulder problems, and evaluator of the radiograph. Sex and age (18–49 and 50–63 years) were extracted from personal identification numbers [21], while information on duration of shoulder problems was extracted from the questionnaire before first visit at the department. Duration of shoulder problems was grouped into 3 categories i.e. < 6 months, ≥ 6 to 24 months, and ≥ 24 months. We included evaluator of the radiograph as a covariate to adjust for any systematic difference in the radiographic findings between the two doctors.

### Statistical analyses

If one or two questions from the OSS were left unanswered, we entered the mean value of all the patient’s other answers. If more than two answers were missing, we did not calculate the score [22]. Associations between a low OSS and radiographic findings were analysed using logistic regression [23]. The associations were estimated using crude, partly, and fully adjusted analyses. In partly adjusted analyses, we included sex, age, medical doctor, and duration of patient reported shoulder problems. A priori, we decided that the full model should include only one variable to represent calcification (present of calcification), spurs (lateral spurs), and acromion shape (acromion tilt). The full model therefore included sex, age, medical doctor, duration of patient reported shoulder problems, presence of calcification, lateral spur, acromioclavicular OA, and acromial tilt. Due to collinearity with lateral spur, Bankart/Hill Sachs lesion was excluded from the fully adjusted model. We did not include any of the calcification classifications in the fully adjusted model due to insufficient reliability of the Molés classification and low prevalence of the subtypes in the other classifications [13]. Association between the radiographic findings and low OSS reported several months later can be doubted if OSS changes with time. Therefore, we performed two sensitivity analyses. We excluded patients with radiographs performed more than 1 month before their completion of the questionnaire, and, in a separate analysis, patients with radiographs performed more than 3 months before their completion of the questionnaire. We used STATA 17 (StataCorp LP, College Station, TX, USA).

## Results

We have previously described the flow chart [13]. In brief, we received a questionnaire from 1039 (57.6%) out of 1803 patients registered in our project database. A total of 850 (81.8%) of the 1039 questionnaire respondents had at least one available radiograph of the shoulder. Of these 850 patients, OSS could not be calculated for 25 patients. Thus, the study comprised 825 patients (54.1% female) with a mean age of 48.2 years (*SD* = 8.84). Out of the 825 patients, 50 (5.9%) of the radiographic examinations did not include an outlet view. The median time between radiographic examination and completion of the questionnaire was 9 days (*SD* = 27.1). Male patients most often had a duration of shoulder problems ≥ 24 months, while female patients most often had a duration of shoulder problems ≥ 6 to < 24 months. The median duration of shoulder problems for both male and female patients was 12 months (interquartile range male/female patients 6 to 32/6 to 30 months) (data not shown). The patients had an average OSS of 27.6 (*SD* = 8.5); female patients 25.7 (*SD* = 8.9) and male patients 29.8 (*SD* = 7.6). Overall, 33.2% of the patients had an OSS < 25.

Table 1 shows number and percentages of patients with an OSS < 25 and the crude, partly, and fully adjusted odds ratios (OR) for OSS < 25 points in relation to radiographic findings, sex, age, and duration of shoulder problems. The percentage of patients with OSS < 25 was by far higher among patients with presence of a lateral spur of birdbeak type (66.7%), a Bankart/Hill-Sachs lesion (50.0%), and acromial tilt > 35° (39.2%) than among patients with any of the other examined radiographic findings. The incidence of abovementioned findings was 2.2% (18/825) for lateral spurs, 7.0% (58/825) for Bankart Hill/Sachs lesion, and 30.9% (255/736) for acromial tilt > 35°. A total of 75% of patients with Bankart/Hills-Sachs lesion had a lateral spur as opposed to 6% among patients without signs of a Bankart/Hills-Sachs lesion (results not shown).

**Table 1:** Percentages of OSS < 25 and crude (*OR*_crude_), partly (*OR*_partly adjusted_), and fully adjusted odds ratios (*OR*_fully adjusted_) in relation to sex, age groups, duration of shoulder problems, evaluator of the radiograph and radiographic findings; *n* = 825

	*n*	Cases		%	*OR*_crude_		95% CI	*OR*_partly adjusted_	95% CI	*OR*_fully adjusted_	95% CI
Calcification present											
No	623	196		31.5	1.00			1.00		1.00	
Yes	202	78		38.6	1.37		0.99; 1.91	1.18	0.83; 1.70	1.28	0.88 1.88
Calcification area											
No calcification	623	196		31.5	1.00			1.00			
≤ 0.2 cm^2^	58	22		37.9	1.33		0.76; 2.32	1.12	0.63; 2.01		
> 0.2 to ≤ 0.6 cm^2^	71	29		40.9	1.50		0.91; 2.49	1.38	0.81; 2.34		
> 0.6 cm^2^	73	27		37.0	1.28		0.77; 2.12	1.05	0.60; 1.84		
Molés classification of calcification											
No calcification	623	196		31.5	1.00			1.00			
Types A and B	44	14		31.8	1.02		0.53; 1.96	0.94	0.47; 1.89		
Type C	23	6		26.1	0.77		0.30; 1.98	0.80	0.30; 2.12		
Type D	135	58		43.0	1.64		1.12; 2.40	1.35	0.89; 2.03		
Acromial type											
I flat	105	31		29.5	1.00			1.00			
II curved	510	168		32.9	1.17		0.74; 1.85	1.13	0.70; 1.83		
III hooked	133	59		36.8	1.39		0.81;2.41	1.39	0.78;2.47		
Lateral spur present											
No	733	232		31.7	1.00			1.00		1.00	
Yes	92	42		45.7	1.81		1.17; 2.81	1.98	1.24; 3.17	2.24	1.35; 3.69
Lateral spur type											
No lateral spur	733	232		31.7	1.00			1.00			
Heel type	36	13		36.1	1.22		0.61; 2.45	1.26	0.60; 2.67		
Traction type	38	17		44.7	1.75		0.91; 3.38	1.99	1.00; 3.97		
Birdbeak type	18	12		66.7	4.32		1.60; 11.65	4.92	1.66; 14.60		
Medial spur present											
No	770	256		33.3	1.00			1.00			
Yes	55	18		32.7	0.98		0.55; 1.75	1.07	0.58; 1.94		
Acromioclavicular OA											
No	726	240		33.1	1.00			1.00			
Yes	99	34		34.3	1.06		0.68; 1.65	1.21	0.76; 1.93	1.23	0.74; 2.05
Bankart/Hill-Sachs lesion											
No	767		245	31.9		1.00		1.00			
Yes	58		29	50.0		2.13	1.25; 3.64	2.49	1.38; 4.48		
Acromial tilt											
> 35° to 50.7°	255	100		39.2	1.00			1.00		1.00	
> 30° to 35°	260	82		31.5	0.71		0.50; 1.03	0.75	0.51; 1.09	0.75	0.51; 1.10
13.6° to 30°	221	60		27.2	0.58		0.39; 0.85	0.62	0.41; 0.93	0.62	0.41; 0.94
Acromion index											
0.23 to 0.6	164	48		29.3	1.00			1.00			
> 0.6 to 0.7	379	126		33.3	1.20		0.81; 1.79	1.15	0.76; 1.74		
> 0.7 to 0.96	277	99		35.7	1.34		0.89; 2.04	1.28	0.83; 1.98		
Lateral acromial angle											
50.3° to 80°	222	77		34.7	1.00			1.00			
> 80° to 90°	417	136		32.6	0.91		0.65; 1.29	0.98	0.69; 1.41		
> 90° to 121°	175	59		33.7	0.96		0.63; 1.45	1.03	0.65; 1.61		

In the univariable analyses, the presence of Molés type D (*OR* = 1.64 (95% CI 1.12–2.40)), lateral spur (particularly the birdbeak type (*OR* = 4.32 (95% CI 1.60–11.65))), acromial tilt < 35° (*OR* = 0.58 (95% CI 0.39; 0–85)), and female sex (*OR* = 2.31 (95% CI 1.71–3.13)) were statistically significantly associated with an OSS < 25. Bankart/Hill-Sachs lesion was also statistically significantly associated with an OSS < 25 in univariable analysis (*OR* = 2.13 (95% CI 1.25–3.64)) and when partly adjusting for sex, age, and duration of shoulder problems (*OR* = 2.49 (95% CI 1.38–4.48)). In the full model, the presence of a lateral spur (*OR* = 2.24 (95% CI 1.36–3.71)), acromial tilt < 35° (*OR* = 0.62 (95% CI 0.41–0.94)), and female sex (*OR* = 2.25 (95% CI 1.59–3.18)) were statistically significantly associated with low OSS. In the sensitivity analysis, excluding patients with radiographs performed more than 1 (*N* = 426) and 3 (*N* = 259) months before completion of the questionnaire on OSS, the results did not change the significance of the results (results not shown).

## Discussion

In this study, we found an increased risk of low OSS among patients with lateral spurs (particularly birdbeak type), Bankart/Hill-Sachs lesions, and high acromial tilt (> 35°) in a population of patients referred to a public department of orthopaedic surgery on suspicion of SIS. An association between female sex and low OSS was also found.

### Strengths and limitations

A strength of the study included the use of the validated OSS, which generally has been used to evaluate shoulder pain and disability in several studies [18, 24–27]. The participants were not aware of the radiographic findings before filling out the questionnaire on OSS, and the evaluators of the radiographs were not aware of patients OSS-score at time of evaluation, reducing the risk of differential misclassification. A limitation of the study was that only 25% of the radiographic examinations included all the recommended projections [13], which might induce non-differential misclassification. The coverage of the project database was only around one-third of the eligible patients according to the Danish National Patient Register [13], but missing registration in the project database was primarily explained by practical issues, in particular periods with high workload in outpatient clinics and without project secretaries/nurses in the individual participating departments, which diminishes the risk of selection bias. Non-response analysis showed an even age and sex distribution among non-included patients and we do not have reasons to suspect differential participation in relation to the OSS. Thus, we do not think that selection bias influenced our results. We are not aware of important confounders that we did not consider.

### Associations between radiographic findings and low OSS

Our cohort had a mean OSS of 27.6 which is in level with the findings in recent studies of populations of patients waiting for surgical treatment of SIS where OSS ranged between 18 and 30 points [26–28]. We found no association between the different measures of calcifications (presence, area, and Molés classification) and low OSS, and neither did we find an association between a hooked acromion (type III) and symptoms. High inter- and intra-rater reliabilities were found for both presence of calcification, calcification area, and acromial type, which cannot explain the ambiguous results [13]. As an alternative to the morphological classification of acromion by Bigliani et al. [29], architectural measures have been introduced, including acromial tilt [30, 31], lateral acromial angle [32], and acromion index [33]. We found that an acromial tilt > 35° was associated with a low OSS. This finding is supported by previous findings of a statistically significant higher acromial tilt in a group of 50 patients with SIS (33°) and 50 patients with rotator cuff tears (34°) than in a group of 50 healthy control patients (29°) [34]. Other studies have reported a higher prevalence of rotator cuff tears in groups of patients with a low tilt [10, 30, 35, 36]. Due to the inconsistent results, we are hesitant to put too much weight on our results regarding acromial tilt. Recent studies have suggested the measure of critical shoulder angle (CSA) as a reliable measure to differentiate between different shoulder pathologies [37, 38]. Acromial tilt was included in this study in favour of CSA due to the high reliability of acromial tilt [13], but maybe the two measures should be combined in future research of patient outcome.

A low OSS was associated with lateral spurs and our results draw attention to the lateral spur of birdbeak type as an important radiographic finding among the patients suffering of severe shoulder pain. The lateral spur of birdbeak type has the sharpest appearance and reaches further into the subacromial space, which could perhaps explain the clinical significance of this finding. The finding of a higher percentage of patients with a low OSS among patients with a lateral spur of birdbeak type and among patients with a Bankart/Hill Sachs lesion could indicate that these two findings were somewhat related, but we have not been able to find previous references of this finding. On a more speculative basis however, we find that our theory is strengthened by the tenfold higher prevalence of lateral spurs in patients with Bankart/Hill Sachs lesion compared to patients without Bankart/Hill Sachs lesion. This could be explained by the traction on deltoid muscle offspring on acromion when patients experience a luxation of the glenohumeral joint. Resulting (micro)trauma and subsequent healing process might with time cause the calcification on the lateral aspect of acromion, leaving a radiologic visible “scar” in the form of a lateral spur. Further research into this hypothesis is warranted.

### Generalisability

In our cohort, the inclusion of patients was closely related to the clinical settings we intended to describe, as we included patients referred for orthopaedic evaluation on suspicion of SIS. The participants were comparable with all patients examined in the region regarding age, sex, and orthopaedic department. This implies that the associations between radiographic findings and low OSS could be generalised to other shoulder patients in Denmark as well as other countries with similar health care organization.

## Conclusion

In terms of associations with patient-reported shoulder pain and disability, lateral spurs, with emphasis on birdbeak type, Bankart/Hill-Sachs lesions, and acromial tilt > 35°, seemed clinically important. These radiological findings may help us understand the background for the symptoms of SIS.

## Data Availability

The data that support the findings of this study are not openly available due to reasons of sensitivity and are available from the corresponding author upon reasonable request. Data are located in controlled access data storage at Central Region Denmark.
